# A longitudinal study of how disability has affected survival in Swedish populations across 150 years

**DOI:** 10.1093/eurpub/ckac129.071

**Published:** 2022-10-25

**Authors:** J Junkka, F Namatovu, E Häggström Lundevaller, K Karhina, L Vikström

**Affiliations:** 1 Centre for Demographic and Ageing Research, Umeå University, Umeå, Sweden; 2 Department of Epidemiology and Global Health, Umeå University, Umeå, Sweden; 3 Department of Philosophical, Historical and Religious Studies, Umeå University, Umeå, Sweden

## Abstract

**Background:**

Studies from across the world report that disability jeopardizes people's health and increases the risk of premature death. The trend has been demonstrated in present-day populations but there has been little research about whether disability affected survival in historical populations. Our objective was to identify long-term developments in the relationship between disability and survival.

**Methods:**

We focused on all causes of mortality in ages 25-42 among groups with any type of disability in Swedish populations in the 1800s, 1900s and 2000s. We used Cox proportional regression and longitudinal micro-level data, measuring both relative differences (HRs) and absolute differences (excess LYL) in premature mortality, across time by disability status and sex.

**Results:**

Although the overall mortality declined profoundly in Sweden during the centuries studied, the strong association between disability and premature mortality persisted, generating a significant disability-survival gap that has held since the 1800s. The absolute difference in this gap narrowed only slightly during the 1900s, from excess LYL due to disability for men of 1.67 (CI 0.17-3.44) in the 1800s, to 0.69 (CI 0.54-0.85) in the 2000s, while for women the change was even smaller, from 1.24 (CI -0.46-3.12) to 0.59 (CI 0.43-0.69). However, the relative difference widened, particularly for women, from HR of 2.46 (CI 0.91-6.70) in the 1800s to HR 12.00 (CI 9.88-14.60) in the 2000s. For men we found a change in HR from 2.30 (CI 1.31-4.06) to 8.48 (CI 7.26-9.92).

**Conclusions:**

Our study is unique in providing comprehensive results about how disability has limited survival for more than 150 years. In Sweden, fundamental societal changes and extensive welfare provisions promoting equality in health and social wellbeing of all citizens have not been enough to improve the survival of younger generations with disabilities.

**Key messages:**

• The strong association between disability and premature mortality persisted from the 1800s to the 2000s.

• In Sweden, fundamental societal changes and extensive welfare provisions promoting equality in health have not improved survival of young adults with disabilities.

